# Comprehensive Profiling of Tubby-Like Protein Expression Uncovers Ripening-Related *TLP* Genes in Tomato (*Solanum lycopersicum*)

**DOI:** 10.3390/ijms21031000

**Published:** 2020-02-03

**Authors:** Yaoxin Zhang, Xiaoqing He, Dan Su, Yuan Feng, Haochen Zhao, Heng Deng, Mingchun Liu

**Affiliations:** Key Laboratory of Bio-Resource and Eco-Environment of Ministry of Education, College of Life Sciences, Sichuan University, Chengdu 610065, China; raise.zyx@gmail.com (Y.Z.); tayt6266@gmail.com (X.H.); scusudan@163.com (D.S.); 2017141241192@stu.scu.edu.cn (Y.F.); scdxzhaohc@163.com (H.Z.); denghscu@163.com (H.D.)

**Keywords:** tubby-like proteins, TLPs, family genes, fruit ripening, tomato

## Abstract

Tubby-like proteins (TLPs), which were firstly identified in obese mice, play important roles in male gametophyte development, biotic stress response, and abiotic stress responses in plants. To date, the role of *TLP* genes in fruit ripening is largely unknown. Here, through a bioinformatics analysis, we identified 11 TLPs which can be divided into three subgroups in tomato (*Solanum lycopersicum*), a model plant for studying fruit development and ripening. It was shown that all SlTLPs except SlTLP11 contain both the Tub domain and F-box domain. An expression profiling analysis in different tomato tissues and developmental stages showed that 7 *TLP* genes are mainly expressed in vegetative tissues, flower, and early fruit developmental stages. Interestingly, other 4 *TLP* members (*SlTLP1*, *SlTLP2*, *SlTLP4*, and *SlTLP5*) were found to be highly expressed after breaker stage, suggesting a potential role of these genes in fruit ripening. Moreover, the induced expression of *SlTLP1* and *SlTLP2* by exogenous ethylene treatment and the down expression of the two genes in ripening mutants, further support their putative role in the ripening process. Overall, our study provides a basis for further investigation of the function of TLPs in plant development and fruit ripening.

## 1. Introduction

Tubby-like proteins (TLPs), first identified in obese mice, are ubiquitous in eukaryotes varying from single-celled to multicellular organisms [1,2]. TLPs are characterized by a signature of the C-terminal tubby domain that forms a closed β barrel with 12 anti-parallel strands and a central hydrophobic α helix [3]. In plants, most known TLPs contain not only a conserved C-terminal tubby domain but also a highly conserved F-box domain at their N-terminus, which is different from the high divergence of the N-terminal sequence in animals [4,5,6,7,8].

Tubby-like proteins were implicated as transcription factors by structural-based functional analysis and subcellular localization assays [3,9]. In animals, TLPs are known to play important roles in the maintenance and functioning of neuronal cells during post-differentiation and development. Mutation of tubby genes can lead to adult obesity, insulin resistance, retinal degeneration, and neurosensory loss [1,8,10,11]. Compared with the wide range of cellular functions of animal TLPs, our knowledge on the role and mode of action of plant TLPs remains largely incomplete. In arabidopsis (*Arabidopsis thaliana*), the plant research model, *11 TLPs* were identified and *AtTLP9* was shown to be involved in responses to salt and drought stress [4,12]. Moreover, redundant functions between *AtTLP3* and *AtTLP9* in plants were found in response to ABA and osmotic stress [4]. *AtTLP2* was reported to regulate the biosynthetic process of homogalacturonic acid in the mucus of seed coats [4]. In rice (*Oryza sativa*), 14 *OsTLPs* were identified and an expression profiling analysis showed that *OsTLPs* are differentially expressed in different tissues at distinct developmental stages [2], suggesting that the *OsTLP* family genes may play an important role in different physiological and developmental processes. More recently, *MdTLP* (Tubby-like proteins in *Malus domestica*) family genes were found to be expressed in multiple organs with high levels in roots, stems, and leaves, but low in flowers of apples. Interestingly, the expression of all *MdTLPs* was up-regulated to some extent under abiotic stress, exogenous ABA and H_2_O_2_ treatments in leaves and root, suggesting the role of *MdTLPs* in responses to stress. Indeed, expression of *MdTLP7* was reported to enhance abiotic stress tolerance in arabidopsis [13]. In addition, overexpression of *CaTLP1* in chickpeas was reported to promote tolerance to salt, drought and oxidative stress [14]. These studies suggested that *TLPs* play an important role in stress response in different plant species, but the potential role of *TLPs* in fruit development is largely unknown [15]. 

Tomato (*Solanum lycopersicum*) is not only one of the most important and popular vegetable plants in the world but also a model for fruit development and ripening research [16]. In this study, through genome-wide identification, classification and phylogenetic analysis, we identified 11 *TLP* family genes which can be divided into three subgroups in tomato. An expression profiling analysis by qRT-PCR showed that four *TLP* family genes (*TLP1*, *TLP2*, *TLP4*, and *TLP5)* are specifically expressed during fruit ripening, suggesting a potential role of these genes in fruit ripening. Moreover, the expression of *TLP1* and *TLP2* can be induced by exogenous ethylene treatment and their expression was found to be significantly downregulated in *rin* and *nor* ripening mutants, further supporting their putative role in the tomato ripening process. Overall, our study sheds light on the putative role of TLPs in plant development and fruit ripening. 

## 2. Results

### 2.1. Genome-Wide Identification and Phylogenetic Analysis of TLPs in Tomato

The TLPs in the whole genome of tomato were identified by using the sequences of arabidopsis TLPs as BLAST queries against the tomato genome (ITAG 2.40). Then we used HMMER to verify whether the identified TLPs contain typical Tub domains (PF01167). A total of 11 TLPs were identified in tomato by using these methods. The SlTLPs peptide ranged in length from 249 to 427 amino acids, with a gene length between 750 and 1284 bp. The predicted isoelectric point (PI) values of TLPs are from 9.16 to 9.63 and protein molecular weight (MW) from 27.74 to 47.80 (kDa). Moreover, subcellular localization prediction suggested that most tomato TLPs were located in the nucleus, with exception of SlTLP2 and SlTLP6 which were predicted to be located in chloroplasts and TLP3 was predicted to be located in mitochondria. These sequence characteristics of TLPs are shown in Table 1.

**Figure 1:**
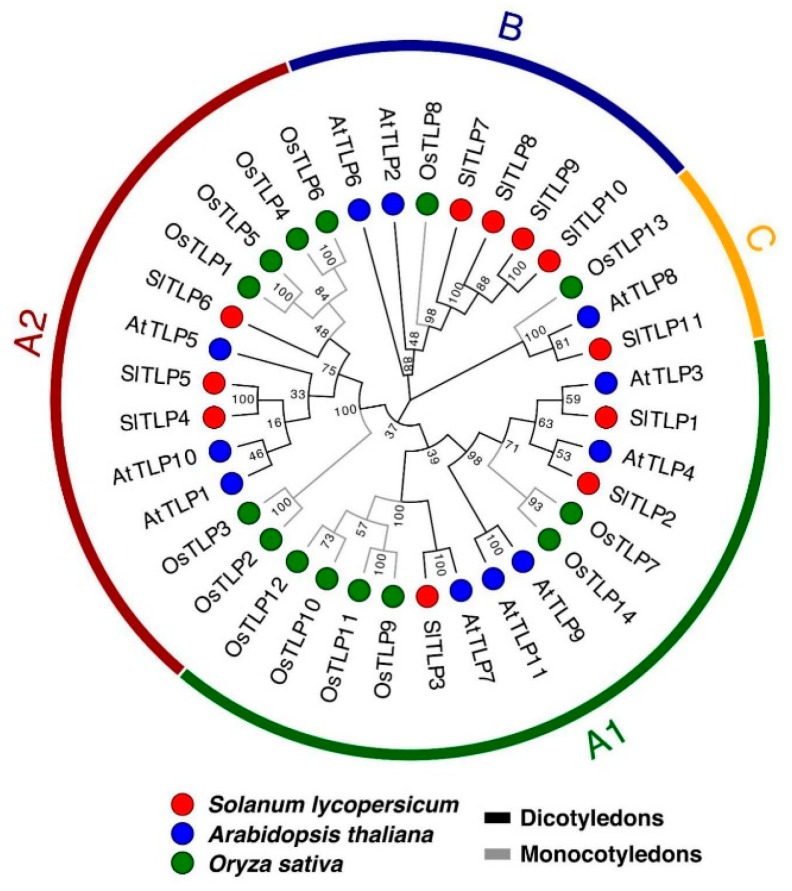
Neighbor-joining (NJ) tree of TLPs in Solanum lycopersicum, Arabidopsis thaliana and Oryza sativa (SlTLPs: TLPs in Solanum lycopersicum; AtTLPs: TLPs in Arabidopsis thaliana; OsTLPs: TLPs in Oryza sativa).

To investigate the phylogenetic relationship of TLP proteins in tomato, we constructed a phylogenetic tree using the neighbor-joining (NJ) method based on multiple sequence alignments of 11 arabidopsis TLP proteins, 14 rice TLP proteins and 11 tomato TLP proteins (Supplementary Table S1). The phylogenetic distribution showed that TLP genes in the three species were all divided into three major clades, A, B and C (Figure 1). Clade A can be further divided into A1 and A2 subgroups. Both subgroup A1 and A2 contained three TLPs proteins in tomato. Clade B contained four tomato TLPs (TLP7, TLP8, TLP9, TLP10) and Clade C only possessed one protein (TLP11). Among the three clades, A and B were closer to each other, while C was estranged. In addition, TLPs in tomato were found to be more similar to that in arabidopsis which is also a dicotyledonous plant.

### 2.2. Motif and Gene Structure Analysis of TLPs in Tomato

**Figure 2:**
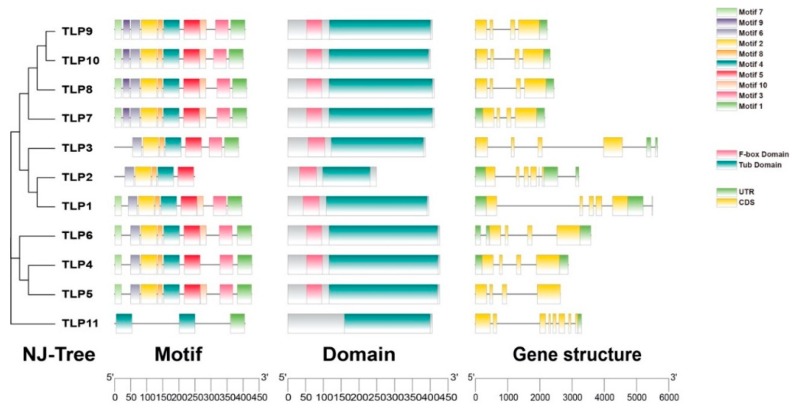
The Motif, domain, and gene structure of TLPs in tomato.

From the Pfam database, we found that the key domain of TLPs in tomato was Tub domain (PF01167) and all TLPs except TLP11 also contain F-box domain (PF00646). To further explore the conservation and diversity of the TLPs, 10 conserved motifs (E ≤ 0.01) were found by MEME (Figure 2 and Supplementary Table S2). All TLPs were found to contain motif 1 and motif 4. Specifically, besides TLP11, all other TLP members contained motif 2, motif 4, motif 5, motif 6, and motif 8. As shown in Figure 2, all *TLP* genes contained both exons and introns. Moreover, the conservation of TLP proteins was higher than that in the gene structure (Figure 2).

### 2.3. Chromosomal Distribution and Selective Pressure Analysis of TLPs in Tomato

To study the distribution of *TLP* genes on chromosomes, we mapped the chromosomal location of tomato *TLP* family genes. The results show that the 11 *TLPs* in tomato were dispersed on seven chromosomes with *TLP2* and *TLP4* located on chromosome 1, *TLP7* and *TLP8* on chromosome 2, *TLP9* and *TLP10* on chromosome 3, *TLP6* and *TLP10* on chromosome 4, *TLP3* on chromosome 7, *TLP1* on chromosome 9, and *TLP5* on chromosome 10.

**Figure 3:**
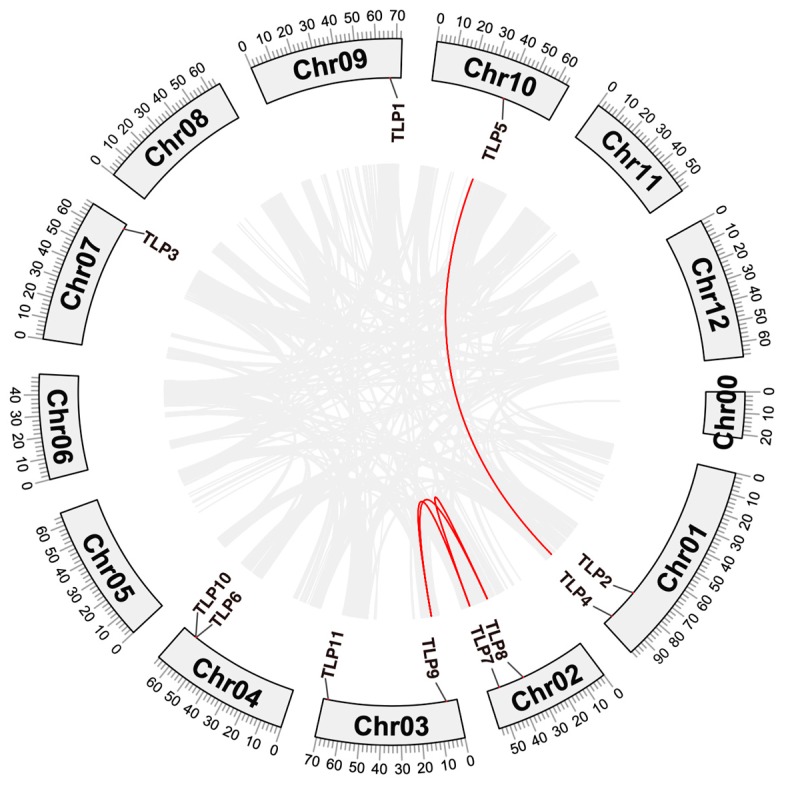
Chromosomal location and homology analysis of *TLPs* in tomato.

To further explore the potential evolutionary mechanism of *TLPs* in tomato, collinear genes in the tomato genome were identified through Blastp and MCScanX. As shown in Figure 3, two groups of genes were found to have strong collinearity. One group was *TLP4* and *TLP5* and another group was *TLP7*, *TLP8*, and *TLP9*. We also calculated their Ka/Ks by MCScanX and found that they are all less than 1 (Ka/Ks: *TLP4-TLP5*, 0.10; *TLP7-TLP8*, 0.20; *TLP7-TLP9*, 0.17; *TLP8-TLP9*, 0.15), which implies that they have strongly purifying selection during evolution.

### 2.4. Analysis of Promoter Sequences of SlTLPs

**Figure 4:**
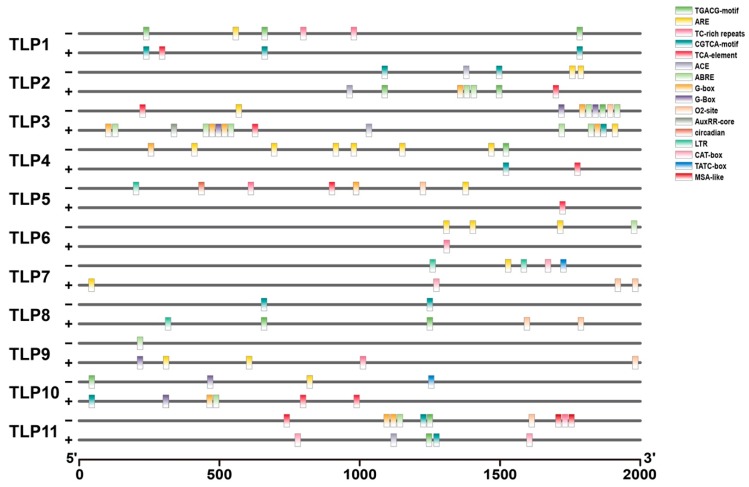
Cis elements in the promoters of *SlTLPs*.

To study the putative role of *TLPs* in tomato, the promoter sequences of tomato *TLPs* were analyzed (CDS upstream 2000 bp) by PlantCare (http://bioinformatics.psb.ugent.be/webtools/plantcare/html/). The cis elements of all *SlTLPs* promoters are shown in Figure 4 and Table 2. Noteworthily, among all *TLP* family genes, *TLP3, TLP10*, and *TLP11* contain a number of different cis elements and *TLP6*, *TLP8*, and *TLP9* contain fewer cis elements. Specifically, most *TLP* promoters contained both CGTCA-motif and TGACG-motif which were related to the jasmonate acid response. Moreover, ARE, which was related to anaerobic reaction and ABRE, which was associated to the abscisic acid response, were found in most *TLPs’* promoters [2,4,6,15]. These results suggest that *TLPs* may play an important role in stress response, but this needs further experimental verification.

### 2.5. Expression Profiling of Tomato TLP Family Genes

**Figure 5:**
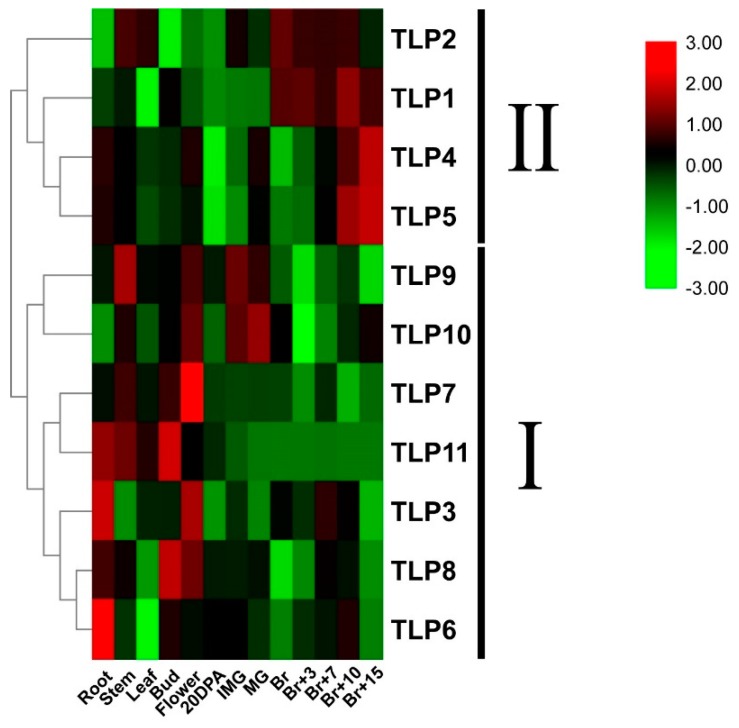
Expression of *TLPs* in different tissues of tomato (20DPA: Fruit at 20 days after anthesis; IMG: Immature green fruit; MG: Mature green fruit; Br: Breaker stage fruit; Br+3: 3 days post-breaker; Br+5: 5 days post-breaker; Br+7: 7 days post-breaker; Br+10: 10-day post-breaker; Br+15: 15 days post-breaker).

To explore the putative function of *TLPs* in tomato, we examined the expression of the 11 *TLPs* in various tissues and different development stages, including the fruit development and ripening process. As shown in Figure 5, based on the expression pattern, the 11 *TLPs* were divided into two subgroups. The *TLPs* in subgroup I (*TLP3, TLP6, TLP7, TLP8, TLP9, TLP10*, and *TLP11*) are mainly expressed in roots, stems, buds, and flower and young fruit which suggests a role of these genes in both vegetative and reproductive development. Interestingly, members of subgroup II (*TLP1, TLP2, TLP4,* and *TLP5*) are highly expressed during the fruit ripening and softening process. More particularly, *TLP1* and *TLP2*, being specifically accumulated from Br (Breaker) to Br+10 (Breaker post 10 days) stages and *TLP4* and *TLP5* are specifically expressed after the Br+10 stage. The specific expression during fruit ripening and softening suggested that *SlTPL1* and *SlTLP2* may play an important role in fruit ripening and *SlTLP4* and *SlTLP5* may be involved in fruit softening.

### 2.6. Expression of TLPs in Fruit Ripening Mutants

**Figure 6:**
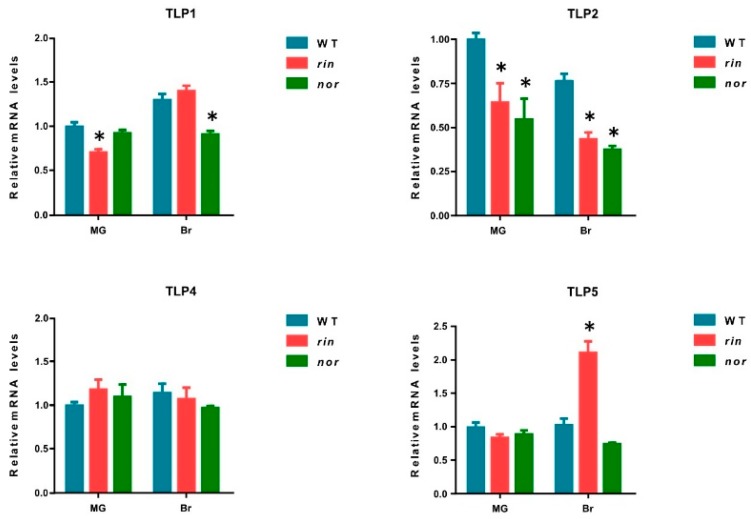
Expression of *TLP1*, *TLP2*, *TLP4*, and *TLP5* in WT, *rin*, and *nor* (MG: mature green fruit; Br: Breaker stage fruit; WT: Wild type; *rin: ripening inhibitor mutant; nor: non-ripening mutant*. The values represent the means of three biological replicates. *, *p* < 0.05 (Student’s *t*-test).).

The role of TLPs in stress resistance has been extensively studied in other plants, while the role of TLPs in fruit ripening remains largely unknown. To further investigate the function of the ripening-related *TLPs* (*TLP1*, *TLP2*, *TLP4*, and *TLP5*) in tomato fruit ripening, we examined the expression levels of *TLP1*, *TLP2*, *TLP4*, and *TLP5* in *ripening-inhibitor* (*rin*) and *non-ripening* (*nor*), two key ripening mutants [17,18]. The results show that *TLP1* is significantly downregulated in *rin* at MG stage, and in *nor* at the Br stage (Figure 6). It is noteworthy that the expression levels of *TLP2* were significantly decreased in both *rin* and *nor* mutants at the MG and Br stages (Figure 6). However, *TLP4* showed no different expression in ripening mutants compared with WT. Interestingly, *TLP5* displayed an upregulation in *rin* at the Br stage. The downregulation of *TLP1* and *TLP2* in ripening mutants further supports the specific role of the two genes in fruit ripening.

### 2.7. Expression of TLPs Under Exogenous Ethylene Treatment

**Figure 7:**
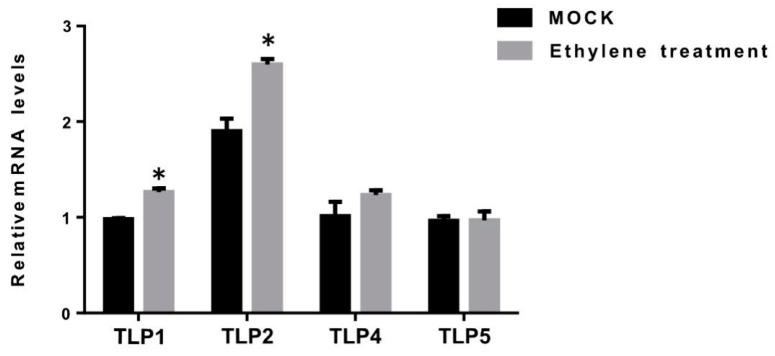
Expression of *TLP1*, *TLP2*, *TLP4*, and *TLP5* under ethylene treatment (MOCK: mature green stage fruit without ethylene treatment; Ethylene treatment: mature green fruit treated with ethylene for 30 min. The values represent the means of three biological replicates. *, *p* < 0.05 (Student’s *t*-test).).

To further investigate the role of *TLPs* in fruit ripening, we investigated the expression of ripening-related *TLPs* (*TLP1, TLP2, TLP4,* and *TLP5*) under exogenous ethylene treatment at MG (mature green) stage fruits (Figure 7). ln line with the potential role of *TLP1* and *TLP2* in fruit ripening, we found that the expression of *TLP1* and *TLP2* was significantly induced with ethylene treatment. In contrast, the expression of *TLP4* and *TLP5* showed no significant change. These results suggest that *TLP1* and *TLP2* may be involved in ethylene-dependent fruit ripening.

## 3. Discussion

Tubby-like proteins (TLPs) have been identified in both animals and plants [15]. In several plant species, TLP family genes were identified and mainly shown to be involved in stress response [2,4,6,7]. However, to date, the TLP family in tomato, one of the most important model plants for fruit ripening research, had not been identified. In this study, to investigate the potential role of TLPs in fruit ripening, we identified 11 TLPs in tomato and showed that two TLP genes, TLP1 and TLP2, may act as ripening regulators based on their specific expression pattern during fruit ripening and their downregulation in ripening mutants.

**Figure 8:**
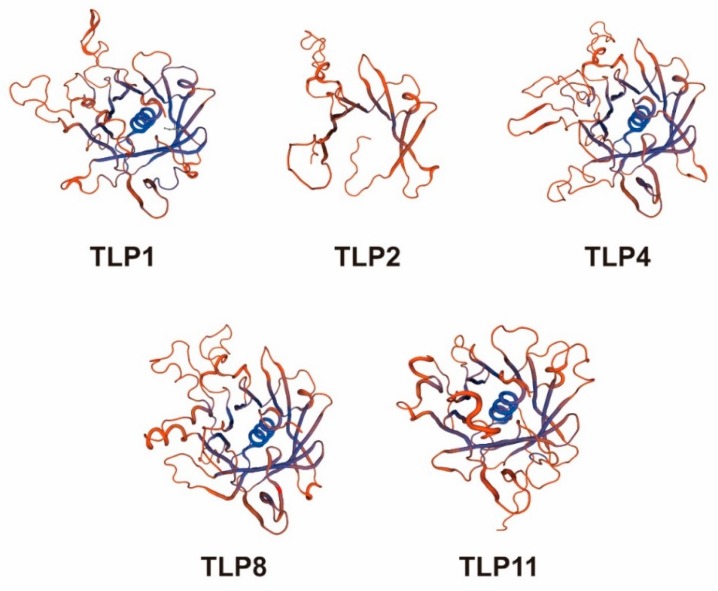
Three-dimensional model of TLPs in tomato.

Based on the analysis of the typical domains and gene structure of TLPs, we found that all TLPs expect TLP11 contain both the Tub domain and F-box domain, which is consistent with previous reports that most plant TLPs contain the F-box domain [2,4,6,7]. Moreover, we found that the motifs in TLP2 are different from other TLPs (Figure 2). To further investigate the difference of structures between TLP2 and other tomato TLP proteins, we built 3D models for Tub domains of TLP1, TLP2, TLP4, TLP8, and TLP11 (Figure 8). From these 3D models, we found that the Tub domain of TLP2 is not complete and it lacks the important part which was thought to be essential for the typical tubby domain (Figure 8). The different structure of TLP2 may suggest a specific role of this gene compared with other TLP genes in tomato. Indeed, the specific expression during fruit ripening and downregulation in ripening mutants of TLPs further supports this hypothesis.

The promoter sequence analysis suggested that most TLPs, especially TLP3, TLP11, and TLP10 in tomato may be related to response to drought and other biotic stresses which were consistent with the function of most TLPs identified in different plant species. Based on the collinear analysis, we found that the TLP4 and TLP5 are paralogs. Moreover, both TLP4 and TLP5 are specific expressed in the late ripening stages. This suggests that paralogs may play similar functions during plant development. Gene expression analysis of tomato TLP genes in different tissues and developmental stages showed that seven genes are mainly expressed in root, stem, flower and young fruit. Interestingly, two genes, TLP1 and TLP2, are found to be highly expressed during fruit ripening, suggesting an important role of the two genes in fruit ripening. Moreover, the downregulation of TLP1 and TLP2 in ripening mutants further supporting the putative role of the two genes in fruit ripening. Overall, our study provides new insight into the role of TLP family genes in fruit ripening and more studies are required to reveal the role and mode of action of TLP genes in fruit ripening.

## 4. Materials and Methods

### 4.1. Data Collection and Identification

Genome, protein, cDNA sequence, and gene annotation files of tomato were downloaded from the NCBI database (http://www.ncbi.nlm.nih.gov/) and Solanaceae Genomics Network (https://solgenomics.net/) [19].

The HMM of the TLP domain (PF01167) was downloaded from Pfam (http://pfam.xfam.org/), and Hmmsearch (3.2.1) was used to identify all possible protein sequences in the whole genome of tomato [20,21]. We used MEME (5.05) [22] (http://meme-suite.org/tools/meme) and Pfam (32.0) to identify the sequences of each presumed protein sequences of TLPs in tomato. We identified proteins based on the best hit proteins in NCBI-Blastp. The isoelectric point (PI) and molecular weight (MW) of TLPs in tomato were analyzed using Expasy [23] (http://web.expasy.org/compute_pi/). The subcellular localization prediction of TLPs in tomato was based on WoLF PSORT [24] (https://wolfpsort.hgc.jp/).

### 4.2. Analysis of Gene Structure, Chromosome Localization, Conserved Motif, and 3D Model

We used Tbtools [25] to draw the gene structure of TLPs in tomato which based on the tomato genome and used the MEME to identify the motif of TLPs in tomato. Full length amino acid sequences of TLPs in tomato were used by the MEME tool [22] (http://meme-suite.org/tools/meme) to identify conserved motifs (Parameter setting: output motifs: 10; minimum motif width: 6; maximum motif width: 200). Based on the tomato genome, we draw the chromosome localization of TLPs in tomato by Circos [26]. SWISS-MODEL [27,28,29] (https://www.swissmodel.expasy.org/) was used for building TLP1, TLP2, TLP4, TLP8, and TLP11 homologous protein model (At least 186 models for each protein were generated using “building model” engine and the best model was selected based on the best global model quality estimation).

### 4.3. Analysis of Collinearity and Selection Pressure

MCScanX [30] was used for collinearity analysis based on the Blast results file which was obtained by Blastp (E < 1e-5) to self-compare the tomato protein. Meanwhile, we used MCScanX to calculate the ka/ks value of the corresponding *TLPs*.

### 4.4. Multiple Sequence Alignment and Phylogenetic Tree Construction

The TLPs in tomato, arabidopsis, and rice were aligncompared by Clustal Omega [31,32] (https://www.ebi.ac.uk/Tools/msa/clustalo/). Neighbor-Joining (NJ) and Maximum likelihood (ML) trees were constructed using MEGA X (10.0.5) [33] with the aligned protein sequences (Bootstrap = 1000 replicates) [34].

### 4.5. Analysis of the Promoter Cis-Regulating Elements

PlantCare [35] (http://bioinformatics.psb.ugent.be/webtools/plantcare/html/) was used to analysis the promoter sequences. 2000 bp of genomic DNA sequence upstream of the transcriptional start sites was obtained from the tomato genome.

### 4.6. Analysis of Gene Expression

The RNA-Seq data of root, stem, leaf, bud, flower, 20DPA, IMG, MG, Br, Br+3, Br+7, Br+10, Br+15 in tomato were downloaded from the TomExpress database [36] (http://tomexpress.toulouse.inra.fr/). The expression data represent normalized counts per base and mean values of multiple cultivars for different tissues and developmental stages and were used to generate heat map representations with R software (https://www.r-project.org). A correlation distance (Spearman) was used to cluster together genes with similar expression profiles

### 4.7. Analysis of Gene Expression in Fruit Ripening Mutants and Ethylene Treatment Fruits

We used qRT-PCR to examine the expression of TLPs in WT, rin, and nor, and also the response of TLPs to exogenous ethylene treatment. cDNA was obtained by reverse transcription according to PrimeScript™RT reagent Kit with gDNA Eraser (Perfect Real Time) (Takara biomedical technology (Beijing) co., LTD., Beijing, China). Real-time quantitative (RT) PCR was performed as described by Pirrello et al., 2006 [37]. Primers for amplification were designed in software PerlPrimer v1.1.21 [38] (Supplementary Table S3). The values represent the means of three biological replicates. *, *p* < 0.05 (Student’s *t*-test).

## Figures and Tables

**Table 1:** Basic Information of tubby-like proteins (TLPs) in tomato.

Group	Name	Locus	Chr	Start	End	Strand	pI	Mw (kDa)	Protein (aa)	ORF (bp)	Subcellular Localization
A1	TLP1	Solyc09g074510	Chr09	66738841	66738900	+	9.33	44.42	396	1191	nucl
	TLP2	Solyc01g067680	Chr01	76374360	76375015	-	9.33	27.74	249	750	chlo
	TLP3	Solyc07g062390	Chr07	65290508	65294312	+	9.16	43.04	386	1161	mito
A2	TLP4	Solyc01g104670	Chr01	92988825	92989308	-	9.35	47.80	427	1284	nucl
	TLP5	Solyc10g046970	Chr10	38906011	38906513	-	9.62	47.80	426	1281	nucl
	TLP6	Solyc04g071440	Chr04	58509459	58510657	+	9.54	47.60	426	1281	chlo
B	TLP7	Solyc02g085130	Chr02	48750167	48750836	+	9.63	46.20	411	1236	nucl
	TLP8	Solyc02g062670	Chr02	34946438	34947426	+	9.25	46.25	411	1236	nucl
	TLP9	Solyc03g033980	Chr03	5712189	5713153	+	9.39	45.52	406	1221	nucl
	TLP10	Solyc04g071750	Chr04	58798600	58798766	+	9.46	44.80	400	1203	nucl
C	TLP11	Solyc03g117730	Chr03	68266827	68267351	-	9.26	45.82	406	1221	nucl

**Table 2:** Cis-acting regulatory elements in the promoter sequences of tomato *TLP* genes.

Name	MeJA		Anaerobic	Light		ABA	SAL	Zein Metabolism	Defense and Stress	Cold	Meristem	Cell Cycle	Gibberellin	Auxin	Circadian Control	Total
	CGTCA	TGACG	ARE	ACE	G-Box	ABRE	TCA	O2-Site	TC-Rich Repeats	LTR	CAT-Box	MSA-Like	TATC-Box	AuxRR-Core	Circadian	
TLP1	3	3	1	0	0	0	1	0	2	0	0	0	0	0	0	10
TLP2	2	2	2	2	1	2	1	0	0	0	0	0	0	0	0	12
TLP3	1	1	2	0	7	7	2	1	0	0	0	0	0	1	0	22
TLP4	1	1	6	0	1	1	1	0	0	0	0	0	0	0	0	11
TLP5	0	0	1	0	1	1	2	1	1	1	0	0	0	0	1	9
TLP6	0	0	3	0	0	1	0	0	1	0	0	0	0	0	0	5
TLP7	0	0	2	0	0	0	0	2	0	2	2	0	1	0	0	9
TLP8	2	2	0	0	0	0	0	2	0	1	0	0	0	0	0	7
TLP9	0	0	2	0	1	1	0	1	1	0	0	0	0	0	0	6
TLP10	1	1	1	0	4	3	2	0	0	0	0	0	1	0	0	13
TLP11	2	2	0	1	2	1	1	1	1	0	2	2	0	0	0	15
Total	12	12	20	3	17	17	10	8	6	4	4	2	2	1	1	119

## Supplementary Materials

Supplementary materials can be found at https://www.mdpi.com/1422-0067/21/3/1000/s1.

## Abbreviations

TLPs	Tubby-like proteins
WT	Wild-type Ailsa Craig
*rin*	Ripening inhibitor mutant
*nor*	Non-ripening mutant
SlTLPs	Tubby-like proteins in *Solanum lycopersicum* (Tomato)
AtTLPs	Tubby-like proteins in *Arabidopsis thaliana* (Arabidopsis)
OsTLPs	Tubby-like proteins in *Oryza sativa* (Rice)
MdTLPs	Tubby-like proteins in *Malus domestica* (Apple)
CaTLPs	Tubby-like proteins in *Cicer arietinum* (Chickpeas)
20DPA	Tomato fruit 20 days after anthesis
IMG	Immature green fruit
MG	Mature green fruit
Br	Breaker stage fruit
Br+3	3 d post-breaker
Br+5	5 d post-breaker
Br+7	7 d post-breaker
Br+10	10 d post-breaker
Br+15	15 d post-breaker
